# μ-Succinato-bis­[aqua­(2,2′:6′,2′′-terpyridine)copper(II)] dinitrate dihydrate

**DOI:** 10.1107/S1600536810006811

**Published:** 2010-02-27

**Authors:** Chongzhen Mei, Qingduo Lei, Peng Zhang

**Affiliations:** a Institute of Environmental and Municipal Engineering, North China University of Water Conservancy and Electric Power, Zhengzhou 450011, People’s Republic of China

## Abstract

The title compound, [Cu_2_(C_4_H_4_O_4_)(C_15_H_11_N_3_)_2_(H_2_O)_2_](NO_3_)_2_·2H_2_O, was synthesized under hydro­thermal conditions. The dinuclear copper complex is located on a crystallographic inversion centre. The Cu^II^ ion is penta­coordinated in a tetra­gonal–pyramidal geometry, with one O atom of a succinate dianion and three N atoms of a 2,2′:6′,2′′-terpyridine ligand occupying the basal plane, and a water O atom located at the apical site. In the crystal structure, O—H⋯O hydrogen bonding links the mol­ecules into a chain parallel to the *a* axis.

## Related literature

For background to the use of saturated aliphatic carboxyl­ate ligands in the preparation of metal-organic complexes, see: Brusau *et al.* (2000 ▶); Rastsvetaeva *et al.* (1996 ▶). For related structures, see: Li *et al.* (2009 ▶); Ke *et al.* (2009 ▶); Jin *et al.* (2008 ▶); He & Huang (2008 ▶); He *et al.* (2007 ▶); Duangthongyou & Siripaisarnpipat (2008 ▶); Liu (2009 ▶); Ng (1998 ▶). 
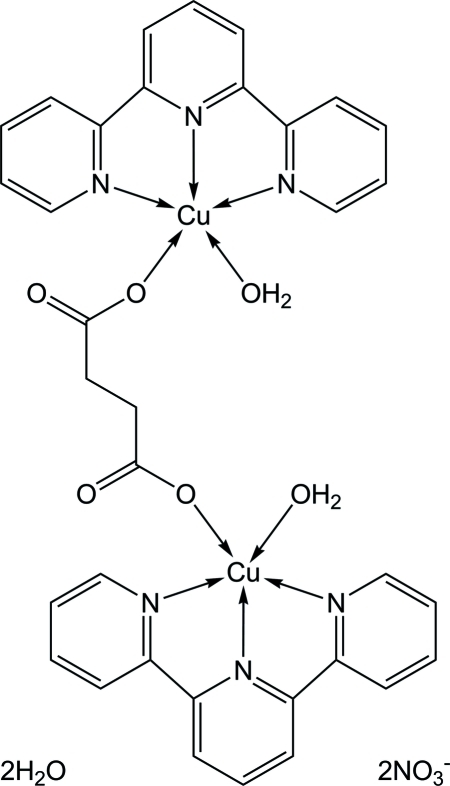


## Experimental

### 

#### Crystal data

[Cu_2_(C_4_H_4_O_4_)(C_15_H_11_N_3_)_2_(H_2_O)_2_](NO_3_)_2_·2H_2_O
*M* *_r_* = 905.77
Triclinic, 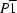

*a* = 7.397 (4) Å
*b* = 10.650 (5) Å
*c* = 12.574 (6) Å
α = 70.196 (9)°
β = 83.512 (9)°
γ = 83.836 (10)°
*V* = 923.5 (8) Å^3^
*Z* = 1
Mo *K*α radiation
μ = 1.23 mm^−1^
*T* = 296 K
0.34 × 0.32 × 0.28 mm

#### Data collection

Bruker SMART APEXII CCD area-detector diffractometer
Absorption correction: multi-scan (*SADABS*; Bruker, 2005 ▶) *T*_min_ = 0.679, *T*_max_ = 0.724
4998 measured reflections
3211 independent reflections
2951 reflections with *I* > 2σ(*I*)
*R*_int_ = 0.096

#### Refinement

*R*[*F* ^2^ > 2σ(*F* ^2^)] = 0.052
*wR*(*F* ^2^) = 0.137
*S* = 0.98
3211 reflections
263 parameters
H-atom parameters constrained
Δρ_max_ = 0.70 e Å^−3^
Δρ_min_ = −0.67 e Å^−3^

### 

Data collection: *APEX2* (Bruker, 2005 ▶); cell refinement: *SAINT* (Bruker, 2005 ▶); data reduction: *SAINT*; program(s) used to solve structure: *SHELXS97* (Sheldrick, 2008 ▶); program(s) used to refine structure: *SHELXL97* (Sheldrick, 2008 ▶); molecular graphics: *SHELXTL* (Sheldrick, 2008 ▶); software used to prepare material for publication: *SHELXTL*.

## Supplementary Material

Crystal structure: contains datablocks I, global. DOI: 10.1107/S1600536810006811/kj2141sup1.cif

Structure factors: contains datablocks I. DOI: 10.1107/S1600536810006811/kj2141Isup2.hkl

Additional supplementary materials:  crystallographic information; 3D view; checkCIF report

## Figures and Tables

**Table 1:** Selected bond lengths (Å)

Cu1—O1	1.917 (2)
Cu1—N3	1.937 (3)
Cu1—N4	2.038 (3)
Cu1—N2	2.049 (3)
Cu1—O1*W*	2.260 (2)

**Table 2:** Hydrogen-bond geometry (Å, °)

*D*—H⋯*A*	*D*—H	H⋯*A*	*D*⋯*A*	*D*—H⋯*A*
O2*W*—H2*WB*⋯O1^i^	0.85	2.33	3.101 (4)	150
O2*W*—H2*WA*⋯O3^ii^	0.85	2.32	3.138 (7)	162
O1*W*—H1*WB*⋯O2*W*	0.85	1.98	2.831 (4)	174
O1*W*—H1*WA*⋯O2^iii^	0.85	1.92	2.755 (3)	167

Symmetry codes: (i) 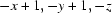; (ii) 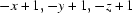; (iii) 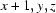.

**Fig. 1.**
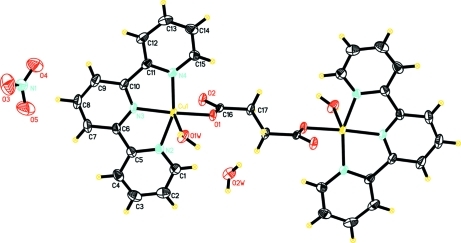
The molecular structure of the title compound with the atom-labelling scheme. Displacement ellipsoids are drawn at the 30% probability level.

**Fig. 2.**
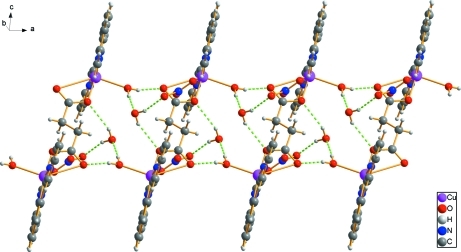
Crystal packing of the title complound. Hydrogen-bond interactions are drawn with dashed lines.

## supplementary crystallographic information

### Comment

As an important family of multidentate O-donor ligands, saturated aliphatic carboxylate ligands have been extensively employed in the preparation of metal-organic complexes (Duangthongyou & Siripaisarnpipat, 2008; He & Huang, 2008; Jin *et al.*, 2008; Li *et al.*, 2009; Liu, 2009; Ke *et al.*,  2009). The succinate dianion has been used as a bridging ligand in the preparation of multinuclear metal complexes. A variety of bridging modes have been found (Ng,1998; Rastsvetaeva *et al.*, 1996; Brusau *et al.*, 2000; He *et al.*, 2007). We report herein the synthesis and crystal stucture of a new succinate complex [Cu_2_(C_4_H_4_O_4_)(C_15_H_11_N_3_)_2_(H_2_O)_2_](NO_3_)_2_.2H_2_O.

In the centrosymmetric dinuclear copper complex (Fig. 1) each of the Cu^II^ ions is pentacoordinated, with one O atom of a succinate dianion and three N atoms of a 2,2':6',2''-terpyridine ligand occupying the basal plane, and a water O atom completing the square-pyramidal geometry from the apical site (Fig. 1). The atoms N2, N3, N4 and O1 are nearly coplanar, with the maximum deviation from the least-squares plane of 0.0292 (13) Å. The Cu atom is displaced by 0.1281 (11) Å from this plane towards the apical O atom.

With O—H···O hydrogen bonds between the coordinated water molecule and the carboxylate group, (Table 1), a one-dimensional chain running parallel to the *a* axis is formed as shown in Fig.2. The uncoordinated water provides an extra link and thereby strengthens the chain and also forms a link to the nitrate counterions.

### Experimental

The title complound was synthesized hydrothermally in a teflon-lined autoclave (25 ml) by heating a mixture of succinic acid (0.2 mmol), 2,2':6',2''-terpyridine (0.4 mmol), Cu(NO_3_)_2_.4H_2_O (0.2 mmol) and Et_3_N (1 ml) in water (10 ml) at 393 K for 3 days. The autoclave was slowly cooled to room temperature. Crystals suitable for X-ray analysis were obtained directly from the reaction product.

### Refinement

The positions of the water H atoms, obtained from a difference Fourier map, were constrained to ideal water geometry and fixed in the final stages of refinement (O—H 0.85 Å), All other H atoms were included in calculated positions, with C—H bond lengths fixed at 0.97 Å (methylene —CH2—) or 0.93Å (aryl group) and were refined in the riding-model approximation. *U*_iso_(H) values were calculated at 1.2 *U*_eq_(C, O).

### Crystal data

**Table:**

[Cu_2_(C_4_H_4_O_4_)(C_15_H_11_N_3_)_2_(H_2_O)_2_](NO_3_)_2_·2H_2_O	*Z* = 1
*M**_r_* = 905.77	*F*(000) = 464
Triclinic, *P*1	*D*_x_ = 1.629 Mg m^−^^3^
Hall symbol: -P 1	Mo *K*α radiation, λ = 0.71073 Å
*a* = 7.397 (4) Å	Cell parameters from 4421 reflections
*b* = 10.650 (5) Å	θ = 3.3–28.0°
*c* = 12.574 (6) Å	µ = 1.23 mm^−^^1^
α = 70.196 (9)°	*T* = 296 K
β = 83.512 (9)°	Block, colourless
γ = 83.836 (10)°	0.34 × 0.32 × 0.28 mm
*V* = 923.5 (8) Å^3^	

### Data collection

**Table:**

Bruker SMART APEXII CCD area-detector diffractometer	3211 independent reflections
Radiation source: fine-focus sealed tube	2951 reflections with *I* > 2σ(*I*)
graphite	*R*_int_ = 0.096
phi and ω scans	θ_max_ = 25.0°, θ_min_ = 1.7°
Absorption correction: multi-scan (*SADABS*; Bruker, 2005)	*h* = −8→8
*T*_min_ = 0.679, *T*_max_ = 0.724	*k* = −12→11
4998 measured reflections	*l* = −14→14

### Refinement

**Table:**

Refinement on *F*^2^	Secondary atom site location: difference Fourier map
Least-squares matrix: full	Hydrogen site location: inferred from neighbouring sites
*R*[*F*^2^ > 2σ(*F*^2^)] = 0.052	H-atom parameters constrained
*wR*(*F*^2^) = 0.137	*w* = 1/[σ^2^(*F*_o_^2^) + (0.1057*P*)^2^ + 0.320*P*] where *P* = (*F*_o_^2^ + 2*F*_c_^2^)/3
*S* = 0.98	(Δ/σ)_max_ < 0.001
3211 reflections	Δρ_max_ = 0.70 e Å^−^^3^
263 parameters	Δρ_min_ = −0.67 e Å^−^^3^
0 restraints	Extinction correction: *SHELXL97* (Sheldrick, 2008), F_c_^*^=kF_c_[1+0.001xF_c_^2^λ^3^/sin(2θ)]^-1/4^
Primary atom site location: structure-invariant direct methods	Extinction coefficient: 0.086 (7)

### Special details

**Table:**

Geometry. All esds (except the esd in the dihedral angle between two l.s. planes) are estimated using the full covariance matrix. The cell esds are taken into account individually in the estimation of esds in distances, angles and torsion angles; correlations between esds in cell parameters are only used when they are defined by crystal symmetry. An approximate (isotropic) treatment of cell esds is used for estimating esds involving l.s. planes.
Refinement. Refinement of *F*^2^ against ALL reflections. The weighted *R*-factor wR and goodness of fit *S* are based on *F*^2^, conventional *R*-factors *R* are based on *F*, with *F* set to zero for negative *F*^2^. The threshold expression of *F*^2^ > σ(*F*^2^) is used only for calculating *R*-factors(gt) etc. and is not relevant to the choice of reflections for refinement. *R*-factors based on *F*^2^ are statistically about twice as large as those based on *F*, and *R*- factors based on ALL data will be even larger.

### Fractional atomic coordinates and isotropic or equivalent isotropic displacement parameters (Å^2^)

**Table:**

	*x*	*y*	*z*	*U*_iso_*/*U*_eq_	
Cu1	0.24585 (4)	0.64732 (3)	0.22948 (2)	0.0286 (2)	
O1W	0.5317 (3)	0.5692 (3)	0.1875 (2)	0.0493 (6)	
H1WA	0.6382	0.5971	0.1717	0.059*	
H1WB	0.5296	0.5065	0.1603	0.059*	
N2	0.2116 (3)	0.4762 (3)	0.3654 (2)	0.0341 (6)	
N3	0.2929 (3)	0.7096 (3)	0.3510 (2)	0.0334 (6)	
N4	0.2612 (4)	0.8463 (3)	0.1423 (2)	0.0356 (6)	
C1	0.1650 (5)	0.3580 (4)	0.3636 (3)	0.0448 (8)	
H1	0.1450	0.3499	0.2946	0.054*	
C2	0.1462 (5)	0.2482 (4)	0.4615 (4)	0.0540 (9)	
H2	0.1150	0.1674	0.4581	0.065*	
C3	0.1743 (5)	0.2605 (4)	0.5637 (3)	0.0573 (10)	
H3	0.1612	0.1882	0.6304	0.069*	
C4	0.2222 (5)	0.3814 (4)	0.5667 (3)	0.0501 (9)	
H4	0.2422	0.3914	0.6351	0.060*	
C5	0.2399 (4)	0.4875 (3)	0.4657 (3)	0.0371 (7)	
C6	0.2890 (4)	0.6223 (3)	0.4581 (3)	0.0360 (7)	
C7	0.3259 (5)	0.6642 (4)	0.5456 (3)	0.0495 (9)	
H7	0.3256	0.6049	0.6195	0.059*	
C8	0.3633 (5)	0.7967 (5)	0.5210 (3)	0.0558 (10)	
H8	0.3883	0.8260	0.5793	0.067*	
C9	0.3642 (5)	0.8862 (4)	0.4107 (4)	0.0516 (9)	
H9	0.3898	0.9747	0.3942	0.062*	
C10	0.3253 (4)	0.8388 (3)	0.3258 (3)	0.0381 (7)	
C11	0.3088 (4)	0.9175 (3)	0.2045 (3)	0.0378 (7)	
C12	0.3356 (5)	1.0526 (3)	0.1567 (3)	0.0509 (9)	
H12	0.3709	1.0998	0.2001	0.061*	
C13	0.3088 (5)	1.1167 (4)	0.0423 (4)	0.0569 (10)	
H13	0.3267	1.2072	0.0083	0.068*	
C14	0.2558 (5)	1.0446 (4)	−0.0195 (3)	0.0526 (9)	
H14	0.2342	1.0863	−0.0953	0.063*	
C15	0.2352 (5)	0.9106 (3)	0.0317 (3)	0.0427 (8)	
H15	0.2020	0.8621	−0.0113	0.051*	
O1	0.1725 (3)	0.5935 (2)	0.11084 (18)	0.0346 (5)	
O2	−0.1129 (3)	0.6332 (2)	0.17197 (19)	0.0418 (5)	
C16	−0.0007 (4)	0.5965 (3)	0.1065 (2)	0.0288 (6)	
C17	−0.0602 (4)	0.5554 (3)	0.0128 (3)	0.0388 (7)	
H17A	−0.1834	0.5272	0.0337	0.047*	
H17B	−0.0632	0.6329	−0.0556	0.047*	
N1	0.1320 (5)	0.8852 (4)	0.7310 (3)	0.0583 (9)	
O3	0.2712 (7)	0.9214 (6)	0.7484 (4)	0.1279 (18)	
O4	0.0270 (5)	0.9562 (4)	0.6617 (3)	0.0940 (12)	
O5	0.1130 (8)	0.7657 (4)	0.7705 (4)	0.1219 (17)	
O2W	0.5518 (4)	0.3538 (3)	0.1018 (3)	0.0623 (7)	
H2WA	0.6184	0.2825	0.1302	0.075*	
H2WB	0.6029	0.3963	0.0374	0.075*	

### Atomic displacement parameters (Å^2^)

**Table:**

	*U*^11^	*U*^22^	*U*^33^	*U*^12^	*U*^13^	*U*^23^
Cu1	0.0298 (3)	0.0333 (3)	0.0296 (3)	0.00097 (16)	−0.00715 (15)	−0.01870 (17)
O1W	0.0279 (11)	0.0587 (15)	0.0753 (16)	0.0008 (10)	0.0012 (11)	−0.0434 (13)
N2	0.0310 (13)	0.0389 (13)	0.0353 (13)	0.0019 (11)	−0.0049 (10)	−0.0166 (11)
N3	0.0310 (13)	0.0439 (14)	0.0343 (12)	0.0067 (11)	−0.0098 (10)	−0.0255 (11)
N4	0.0366 (14)	0.0353 (13)	0.0397 (13)	0.0043 (11)	−0.0072 (11)	−0.0193 (11)
C1	0.0395 (17)	0.0459 (18)	0.0524 (19)	−0.0009 (15)	−0.0061 (14)	−0.0205 (15)
C2	0.0411 (19)	0.0442 (19)	0.071 (2)	−0.0029 (16)	−0.0040 (17)	−0.0116 (17)
C3	0.0428 (19)	0.059 (2)	0.053 (2)	0.0043 (17)	0.0019 (16)	−0.0007 (17)
C4	0.0449 (19)	0.065 (2)	0.0345 (16)	0.0085 (17)	−0.0017 (14)	−0.0136 (16)
C5	0.0278 (15)	0.0488 (18)	0.0330 (14)	0.0087 (13)	−0.0025 (11)	−0.0149 (13)
C6	0.0273 (15)	0.0522 (18)	0.0340 (15)	0.0081 (13)	−0.0058 (11)	−0.0233 (13)
C7	0.0391 (18)	0.080 (3)	0.0383 (17)	0.0101 (18)	−0.0097 (14)	−0.0334 (17)
C8	0.046 (2)	0.084 (3)	0.060 (2)	0.0086 (19)	−0.0143 (17)	−0.054 (2)
C9	0.0449 (19)	0.060 (2)	0.069 (2)	0.0026 (17)	−0.0116 (17)	−0.0462 (19)
C10	0.0298 (15)	0.0463 (18)	0.0511 (18)	0.0047 (13)	−0.0087 (13)	−0.0334 (15)
C11	0.0284 (15)	0.0378 (16)	0.0547 (19)	0.0052 (12)	−0.0076 (13)	−0.0262 (14)
C12	0.047 (2)	0.0402 (18)	0.075 (3)	0.0030 (16)	−0.0072 (18)	−0.0330 (18)
C13	0.050 (2)	0.0347 (18)	0.080 (3)	0.0070 (16)	−0.0016 (19)	−0.0162 (18)
C14	0.045 (2)	0.050 (2)	0.054 (2)	0.0114 (17)	−0.0087 (16)	−0.0086 (16)
C15	0.0404 (17)	0.0430 (18)	0.0440 (17)	0.0064 (14)	−0.0092 (14)	−0.0145 (14)
O1	0.0289 (11)	0.0497 (13)	0.0371 (11)	0.0008 (9)	−0.0093 (8)	−0.0286 (10)
O2	0.0328 (11)	0.0611 (14)	0.0450 (12)	0.0029 (10)	−0.0050 (9)	−0.0364 (11)
C16	0.0299 (14)	0.0288 (13)	0.0329 (14)	0.0023 (11)	−0.0079 (11)	−0.0165 (11)
C17	0.0298 (15)	0.0524 (19)	0.0490 (17)	0.0093 (14)	−0.0127 (13)	−0.0368 (15)
N1	0.063 (2)	0.059 (2)	0.0496 (17)	0.0021 (17)	0.0010 (16)	−0.0169 (15)
O3	0.120 (4)	0.167 (5)	0.118 (3)	−0.050 (3)	−0.041 (3)	−0.054 (3)
O4	0.083 (3)	0.089 (3)	0.100 (3)	0.016 (2)	−0.025 (2)	−0.020 (2)
O5	0.164 (5)	0.074 (3)	0.105 (3)	−0.018 (3)	−0.020 (3)	0.005 (2)
O2W	0.0656 (17)	0.0518 (15)	0.0742 (18)	0.0006 (13)	−0.0035 (14)	−0.0290 (14)

### Geometric parameters (Å, °)

**Table:**

Cu1—O1	1.917 (2)	C8—C9	1.391 (6)
Cu1—N3	1.937 (3)	C8—H8	0.9300
Cu1—N4	2.038 (3)	C9—C10	1.394 (5)
Cu1—N2	2.049 (3)	C9—H9	0.9300
Cu1—O1W	2.260 (2)	C10—C11	1.483 (5)
O1W—H1WA	0.8501	C11—C12	1.385 (5)
O1W—H1WB	0.8501	C12—C13	1.394 (6)
N2—C5	1.348 (4)	C12—H12	0.9300
N2—C1	1.349 (5)	C13—C14	1.372 (6)
N3—C10	1.345 (4)	C13—H13	0.9300
N3—C6	1.351 (4)	C14—C15	1.370 (5)
N4—C15	1.351 (4)	C14—H14	0.9300
N4—C11	1.354 (4)	C15—H15	0.9300
C1—C2	1.387 (5)	O1—C16	1.285 (4)
C1—H1	0.9300	O2—C16	1.230 (3)
C2—C3	1.376 (6)	C16—C17	1.510 (4)
C2—H2	0.9300	C17—C17^i^	1.503 (6)
C3—C4	1.386 (6)	C17—H17A	0.9700
C3—H3	0.9300	C17—H17B	0.9700
C4—C5	1.389 (5)	N1—O3	1.204 (6)
C4—H4	0.9300	N1—O5	1.217 (5)
C5—C6	1.487 (5)	N1—O4	1.231 (5)
C6—C7	1.383 (5)	O2W—H2WA	0.8499
C7—C8	1.390 (6)	O2W—H2WB	0.8500
C7—H7	0.9300		
			
O1—Cu1—N3	173.78 (9)	C6—C7—H7	120.6
O1—Cu1—N4	98.53 (10)	C8—C7—H7	120.6
N3—Cu1—N4	80.04 (11)	C7—C8—C9	121.1 (3)
O1—Cu1—N2	100.55 (11)	C7—C8—H8	119.5
N3—Cu1—N2	79.94 (12)	C9—C8—H8	119.5
N4—Cu1—N2	158.56 (11)	C8—C9—C10	117.8 (4)
O1—Cu1—O1W	86.91 (9)	C8—C9—H9	121.1
N3—Cu1—O1W	99.30 (10)	C10—C9—H9	121.1
N4—Cu1—O1W	100.14 (10)	N3—C10—C9	120.2 (3)
N2—Cu1—O1W	90.58 (10)	N3—C10—C11	112.8 (3)
Cu1—O1W—H1WA	138.1	C9—C10—C11	127.0 (3)
Cu1—O1W—H1WB	111.0	N4—C11—C12	121.6 (3)
H1WA—O1W—H1WB	107.7	N4—C11—C10	114.1 (3)
C5—N2—C1	118.7 (3)	C12—C11—C10	124.3 (3)
C5—N2—Cu1	114.3 (2)	C11—C12—C13	118.9 (3)
C1—N2—Cu1	127.0 (2)	C11—C12—H12	120.6
C10—N3—C6	122.5 (3)	C13—C12—H12	120.6
C10—N3—Cu1	118.7 (2)	C14—C13—C12	119.2 (3)
C6—N3—Cu1	118.8 (2)	C14—C13—H13	120.4
C15—N4—C11	118.5 (3)	C12—C13—H13	120.4
C15—N4—Cu1	127.4 (2)	C15—C14—C13	119.3 (4)
C11—N4—Cu1	114.1 (2)	C15—C14—H14	120.3
N2—C1—C2	122.0 (3)	C13—C14—H14	120.3
N2—C1—H1	119.0	N4—C15—C14	122.4 (3)
C2—C1—H1	119.0	N4—C15—H15	118.8
C3—C2—C1	119.0 (4)	C14—C15—H15	118.8
C3—C2—H2	120.5	C16—O1—Cu1	115.18 (17)
C1—C2—H2	120.5	O2—C16—O1	123.0 (3)
C2—C3—C4	119.5 (3)	O2—C16—C17	121.3 (3)
C2—C3—H3	120.2	O1—C16—C17	115.7 (2)
C4—C3—H3	120.2	C17^i^—C17—C16	114.3 (3)
C3—C4—C5	118.7 (4)	C17^i^—C17—H17A	108.7
C3—C4—H4	120.6	C16—C17—H17A	108.7
C5—C4—H4	120.6	C17^i^—C17—H17B	108.7
N2—C5—C4	122.0 (3)	C16—C17—H17B	108.7
N2—C5—C6	114.2 (3)	H17A—C17—H17B	107.6
C4—C5—C6	123.8 (3)	O3—N1—O5	116.3 (5)
N3—C6—C7	119.7 (3)	O3—N1—O4	123.9 (5)
N3—C6—C5	112.7 (3)	O5—N1—O4	118.6 (5)
C7—C6—C5	127.6 (3)	H2WA—O2W—H2WB	107.7
C6—C7—C8	118.7 (3)		
			
O1—Cu1—N2—C5	−174.99 (19)	N2—C5—C6—N3	1.1 (4)
N3—Cu1—N2—C5	−1.29 (19)	C4—C5—C6—N3	−178.3 (3)
N4—Cu1—N2—C5	−22.4 (4)	N2—C5—C6—C7	180.0 (3)
O1W—Cu1—N2—C5	98.0 (2)	C4—C5—C6—C7	0.6 (5)
O1—Cu1—N2—C1	5.0 (3)	N3—C6—C7—C8	1.0 (5)
N3—Cu1—N2—C1	178.7 (3)	C5—C6—C7—C8	−177.8 (3)
N4—Cu1—N2—C1	157.6 (3)	C6—C7—C8—C9	0.0 (5)
O1W—Cu1—N2—C1	−81.9 (3)	C7—C8—C9—C10	0.3 (5)
N4—Cu1—N3—C10	−4.6 (2)	C6—N3—C10—C9	2.7 (5)
N2—Cu1—N3—C10	−176.9 (2)	Cu1—N3—C10—C9	−178.4 (2)
O1W—Cu1—N3—C10	94.1 (2)	C6—N3—C10—C11	−175.6 (2)
N4—Cu1—N3—C6	174.3 (2)	Cu1—N3—C10—C11	3.3 (3)
N2—Cu1—N3—C6	2.0 (2)	C8—C9—C10—N3	−1.6 (5)
O1W—Cu1—N3—C6	−86.9 (2)	C8—C9—C10—C11	176.4 (3)
O1—Cu1—N4—C15	−3.3 (3)	C15—N4—C11—C12	−1.7 (5)
N3—Cu1—N4—C15	−177.1 (3)	Cu1—N4—C11—C12	176.3 (2)
N2—Cu1—N4—C15	−156.0 (3)	C15—N4—C11—C10	177.2 (3)
O1W—Cu1—N4—C15	85.1 (3)	Cu1—N4—C11—C10	−4.8 (3)
O1—Cu1—N4—C11	178.9 (2)	N3—C10—C11—N4	1.2 (4)
N3—Cu1—N4—C11	5.1 (2)	C9—C10—C11—N4	−177.0 (3)
N2—Cu1—N4—C11	26.2 (4)	N3—C10—C11—C12	−179.9 (3)
O1W—Cu1—N4—C11	−92.7 (2)	C9—C10—C11—C12	2.0 (5)
C5—N2—C1—C2	−0.2 (5)	N4—C11—C12—C13	1.4 (5)
Cu1—N2—C1—C2	179.8 (2)	C10—C11—C12—C13	−177.4 (3)
N2—C1—C2—C3	0.6 (5)	C11—C12—C13—C14	0.3 (5)
C1—C2—C3—C4	−0.6 (5)	C12—C13—C14—C15	−1.7 (6)
C2—C3—C4—C5	0.2 (5)	C11—N4—C15—C14	0.3 (5)
C1—N2—C5—C4	−0.1 (4)	Cu1—N4—C15—C14	−177.4 (3)
Cu1—N2—C5—C4	179.9 (2)	C13—C14—C15—N4	1.5 (5)
C1—N2—C5—C6	−179.5 (3)	N4—Cu1—O1—C16	−90.9 (2)
Cu1—N2—C5—C6	0.5 (3)	N2—Cu1—O1—C16	79.3 (2)
C3—C4—C5—N2	0.1 (5)	O1W—Cu1—O1—C16	169.3 (2)
C3—C4—C5—C6	179.5 (3)	Cu1—O1—C16—O2	1.2 (4)
C10—N3—C6—C7	−2.4 (4)	Cu1—O1—C16—C17	179.5 (2)
Cu1—N3—C6—C7	178.7 (2)	O2—C16—C17—C17^i^	−145.5 (4)
C10—N3—C6—C5	176.6 (2)	O1—C16—C17—C17^i^	36.1 (5)
Cu1—N3—C6—C5	−2.3 (3)		

Symmetry codes: (i) −*x*, −*y*+1, −*z*.

### Hydrogen-bond geometry (Å, °)

**Table:**

*D*—H···*A*	*D*—H	H···*A*	*D*···*A*	*D*—H···*A*
O2W—H2WB···O1^ii^	0.85	2.33	3.101 (4)	150
O2W—H2WA···O3^iii^	0.85	2.32	3.138 (7)	162
O1W—H1WB···O2W	0.85	1.98	2.831 (4)	174
O1W—H1WA···O2^iv^	0.85	1.92	2.755 (3)	167

Symmetry codes: (ii) −*x*+1, −*y*+1, −*z*; (iii) −*x*+1, −*y*+1, −*z*+1; (iv) *x*+1, *y*, *z*.
